# Association between Dietary Inflammatory Index, Dietary Patterns, Plant-Based Dietary Index and the Risk of Obesity

**DOI:** 10.3390/nu13051536

**Published:** 2021-05-02

**Authors:** Yoko B. Wang, Nitin Shivappa, James R. Hébert, Amanda J. Page, Tiffany K. Gill, Yohannes Adama Melaku

**Affiliations:** 1 Vagal Afferent Research Group, Adelaide Medical School, University of Adelaide, Adelaide, SA 5000, Australia; yokobrigitte.wang@adelaide.edu.au (Y.B.W.); amanda.page@adelaide.edu.au (A.J.P.); 2 Nutrition, Diabetes & Gut Health, Lifelong Health Theme, South Australian Health and Medical Research Institute (SAHMRI), Adelaide, SA 5000, Australia; 3 Cancer Prevention and Control Program, University of South Carolina, Columbia, SC 29208, USA; shivappa@email.sc.edu (N.S.); jhebert@mailbox.sc.edu (J.R.H.); 4 Department of Epidemiology and Biostatistics, Arnold School of Public Health, University of South Carolina, Columbia, SC 29208, USA; 5 Adelaide Medical School, University of Adelaide, Adelaide, SA 5000, Australia; tiffany.gill@adelaide.edu.au; 6 Adelaide Institute for Sleep Health, College of Medicine and Public Health, Flinders University, Bedford Park, SA 5042, Australia

**Keywords:** dietary inflammatory index, dietary pattern, plant-based diet, diet quality, obesity, prospective study

## Abstract

Evidence on the association between various dietary constructs and obesity risk is limited. This study aims to investigate the longitudinal relationship between different diet indices and dietary patterns with the risk of obesity. Non-obese participants (*n* = 787) in the North West Adelaide Health Study were followed from 2010 to 2015. The dietary inflammatory index (DII^®^), plant-based dietary index (PDI) and factor-derived dietary pattern scores were computed based on food frequency questionnaire data. We found the incidence of obesity was 7.62% at the 5-year follow up. In the adjusted model, results from multivariable log-binomial logistic regression showed that a prudent dietary pattern (RR_Q5_ vs. _Q1_ = 0.38; 95% CI: 0.15–0.96), healthy PDI (RR = 0.31; 95% CI: 0.12–0.77) and overall PDI (RR = 0.56; 95% CI: 0.23–1.33) were inversely associated with obesity risk. Conversely, the DII (RR = 1.59; 95% CI: 0.72–3.50), a Western dietary pattern (RR = 2.16; 95% CI: 0.76–6.08) and unhealthy PDI (RR = 1.94; 95% CI: 0.81–4.66) were associated with increased risk of obesity. Based on the cubic spline analysis, the association between an unhealthy PDI or diet quality with the risk of obesity was non-linear. In conclusion, an anti-inflammatory diet, healthy diet or consumption of healthy plant-based foods were all associated with a lower risk of developing obesity.

## 1. Introduction

Obesity has become a global challenge, with a devastating impact on public health, the economy and climate [1]. In the past four decades, the global prevalence of obesity has rapidly increased. According to the World Health Organization (WHO), 49% of adults globally were overweight, and 13% were obese in 2016 [2]. In 2017–2018, 67% of Australian adults were overweight and 31% were obese [3]. Furthermore, the escalating global prevalence of obesity begins early in life [4]. If this trend continues, it is predicted that 18% of men, over 21% of women, and 124 million children and adolescents in the global population will be obese in 2025 [5]. 

Diet is a key modifiable factor in obesity prevention. There are different dietary indices or patterns of overall dietary intake currently available, such as the dietary inflammatory index (DII^®^), dietary quality indices (e.g., prudent and Western diet), and a recently developed plant-based dietary index (PDI). The DII is a literature-based algorithm scoring tool, designed to measure the inflammatory potential of a diet [6], while the dietary pattern is an emerging analysis approach meant to evaluate the relationship between diet and disease, which generally categorizes diet into a healthy (prudent) and unhealthy (Western) pattern [7]. Conversely, PDI is a recently developed index with a focus on the intake of plant-based foods [8]. 

Many studies have examined the association between DII, dietary patterns or PDI with metabolic syndromes [9,10] or chronic diseases such as cardiovascular diseases [11,12], diabetes [12,13] and certain types of cancers [14]. However, evidence on the association between the DII, PDI or dietary patterns and obesity prevention are limited, particularly in Australia. In fact, the existing studies are mainly cross-sectional [15], conducted in specific populations (e.g., specific age groups or sex) [16,17], and assessed each measurement tool independently. Given that food environment and availability could vary across different regions, and the fact that obesity develops gradually over time, understanding the longitudinal relationship between dietary indices, dietary patterns and the risk of obesity becomes important. Therefore, in this study, we aimed to investigate the prospective association between the DII, dietary patterns and intake of plant-based foods with the risk of obesity in the North West Adelaide Health Study (NWAHS) cohort. To our knowledge, this is the first study to examine the longitudinal association between DII, PDI, dietary patterns and obesity risk in Australia.

## 2. Materials and Methods

### 2.1. Study Design and Population

The NWAHS is a longitudinal cohort study representing one-third of the population in South Australia and approximately half of the metropolitan area (~1.1 million people) [18]. This state has the second highest proportion of elderly residents (≥65 years old) among all the Australian states and territories after Tasmania [18,19]. Recruitment details have been described in detail previously [20]. In brief, eligible participants (age ≥ 18 years old), from northern and western suburbs of Adelaide, South Australia, were randomly selected from the electronic White Page^®^ and recruited using the telephone. Three stages of data collection were conducted in: 1999–2003 (Stage 1), 2004–2006 (Stage 2) and 2008–2010 (Stage 3), using a computer-assisted telephone interview (CATI), self-administered questionnaire and clinic examination. In 2015, a follow up study using a self-completed online or postal survey was conducted (NW15). 

**Figure 1:**
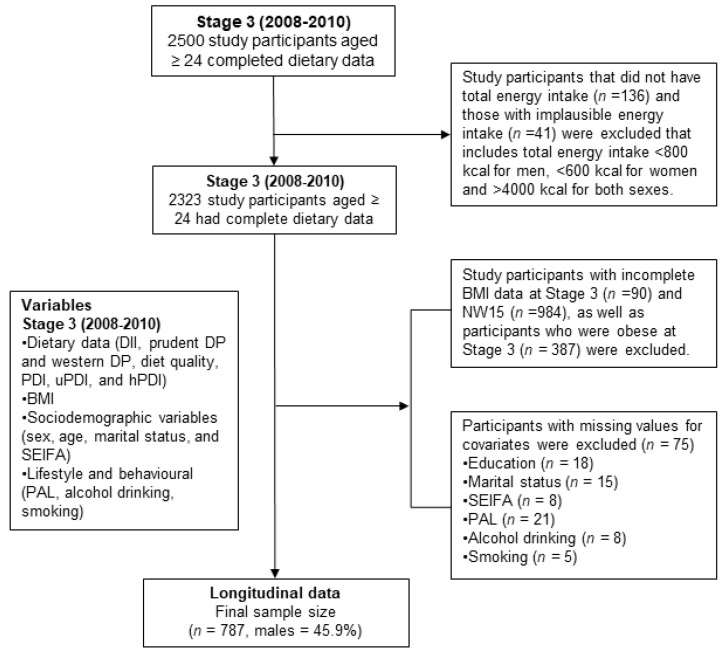
Sampling description of the study participants in the North West Adelaide Health Study (NWAHS). BMI: body mass index; SEIFA: Socio-Economic Indexes for Area; PAL: Physical Level Activity; DII: Dietary Inflammatory Index; DP: dietary pattern; PDI: Plant-based Dietary Index; uPDI: unhealthy Plant-Based Dietary Index; hPDI: healthy Plant-Based Dietary Index.

In this study, we utilised data from Stage 3 and NW15 with a total of 787 participants included for a prospective study examining the association between the DII, dietary pattern or PDI and the risk of obesity (Figure 1). We excluded 177 participants, including participants without total energy intake data (*n* = 136) or if the participants total energy intake was <800 kcal for men, <600 kcal for women, and >4000 kcal for both males and females (*n* = 41). Another 1461 participants were excluded due to lack of BMI data in stage 3 (*n* = 90) or NW15 (*n* = 984) or were obese at stage 3 (*n* = 387). We also excluded 75 due to missing values for covariates.

This study was approved by The Human Ethics Research Committee, Queen Elizabeth Hospital, South Australia. All participants provided written informed consent.

### 2.2. Measures of BMI at Stage 3 and NW15

Height and weight were measured using standard protocols during the clinic examination in Stage 3, while in NW15, height and weight were self-reported. BMI was computed as weight(kg)/height(m)^2^ [21]. Participants with BMI ≥ 30 kg/m^2^ were categorised as obese and participants with BMI < 30 kg/m^2^ were categorised as non-obese. 

### 2.3. Dietary Assessment and Analysis

An assessment of the previous 12 months dietary intake was conducted at Stage 3 using a revised Food Frequency Questionnaire (FFQ), the validated Dietary Questionnaire for Epidemiological Studies Version 3 (DQES-V3.1), developed by Cancer Council Victoria [22]. The questionnaire was self-completed. The data obtained from this questionnaire were used to generate the dietary constructs below: 

### 2.4. Dietary Inflammatory Index

The construction of the DII has been described in detail previously [6]. In brief, the DII is a robust literature-based scoring algorithm that compares the inflammatory properties of diets. Calculation was performed by linking to a representative global database from 11 populations in the world to obtain a z-score. The z-score was then converted into a percentile, followed by doubling the value and subtracted by 1 to centre the values. The overall DII score, for each participant, was obtained from the sum of all food parameters and adjusted article scores. The higher the DII value, the more pro-inflammatory the diet and, conversely, the lower the score the more anti-inflammatory the diet.

The DII value in this study was generated by scoring 29 out of 45 food parameters based on their effect on 6 inflammatory markers (IL-1ß, IL-4, IL-6, IL-10, TNF-α and C-reactive protein). The food parameters included carbohydrate, protein, fat, saturated fatty acids, monounsaturated fatty acids, polyunsaturated fatty acids, cholesterol, trans-fat, alcohol, iron, zinc, thiamine, magnesium, niacin, riboflavin, fibre, omega-3, omega-6, folic acid, β-carotene, tea, garlic, onions, vitamin A, B6, B12, C, D, and E. The DII value was adjusted to the total energy intake.

### 2.5. Dietary Pattern

We used two dietary patterns, the prudent pattern (healthy diet) and the Western pattern (unhealthy diet), which have been identified previously using principal composite analysis (PCA) [23]. In brief, thirty-nine dietary patterns were constructed based on food groups. A scree plot, an eigenvalue (>1) and interpretability were used to determine two retained factors. Varimax rotation was applied to increase factor interpretability. The sum of factor loading coefficients, standardized by the daily intake of individual food item, was used to calculate factor scores for each participant as well as the retained factors. A Kaiser–Meyer–Olkin test was used to check for sample adequacy. As participants are unlikely to exclusively follow either a prudent or Western dietary intake pattern, the Western pattern score was subtracted from the prudent pattern score for each participant to generate a value for “diet quality”.

### 2.6. Plant-Based Dietary Index

We also developed three plant-based dietary indices, i.e., PDI, healthy PDI (hPDI) and unhealthy PDI (uPDI), using the approach described by Satija et al. [24]. Eighteen food groups were generated to represent healthy and unhealthy plant foods, as well as animal foods (Supplementary Table S1). Healthy and unhealthy plant foods were distinguished based on the existing literature on the association between various foods and chronic diseases. We excluded plant foods that could not be categorized as healthy or unhealthy (e.g., alcoholic drinks). Nonetheless, alcohol intake was adjusted for in multivariable regression analyses. Each food group was then categorized into sex-specific deciles and scored from 1 to 10 positively (Q1 = 1 → Q10 = 10) or vice versa (Q1 = 10 → Q10 = 1). For the PDI, we allocated positive scores for healthy and unhealthy plant foods while reverse scores were allocated for animal foods. For the hPDI, healthy plant foods were allocated positive scores while less healthy plant foods and animal foods were given reverse scores. To create the uPDI, we gave positive scores to less healthy plant foods and allocated reverse scores for healthy plant foods and animal foods. The sum of the food scores were used to acquire PDI, uPDI and hPDI values that ranged from 18 to 180. A higher index represents more plant-based and less animal-based diet.

### 2.7. Assessment of Covariates

Potential behavioural and socioeconomic confounders that may be associated with diet factors and the risk of obesity were identified. Detailed criteria to determine categories for each covariate have been described previously [20]. Smoking status was categorised into non-smoker, ex-smoker, and current smoker. Alcohol risk was categorised into non-drinkers and no risk, low risk, intermediate risk, high to very high risk, and incomplete information based on the 1989 National Heart Foundation Risk Factor Prevalence study classification formula [25]. Physical activity levels (PAL) were assessed using the Active Australia Survey and the results were categorised into no activity, insufficient activity, and sufficient activity [26]. 

Socioeconomic status was collected based on the Socio-Economic Index for Areas (SEIFA), developed by the Australian Bureau of Statistics, which ranks areas in Australia according to relative socioeconomic advantage and disadvantage based on five-yearly census data [27]. In this study, we used the Index of Relative Social Disadvantage (IRSD) and divided the determined value into quintiles where the lowest represents the greatest disadvantage. Participants’ marital status was categorised into married or living with partner, separated/divorced, widowed, never married, or not stated.

### 2.8. Statistical Analyses

Descriptive analysis of sociodemographic and lifestyle characteristics was conducted across the factor quintiles. Mean values and standard deviations were calculated for continuous and normally distributed variables. Proportions were calculated for categorical variables. Chi-square tests for categorical variables and ANOVA were used to identify significant differences across different levels of dietary pattern scores. The *p*-value for trend was determined using quintiles as continuous variables. Generalized linear model with binomial family and log link was used to estimate the risk ratio used to assess the association between dietary patterns and obesity. For the dietary patterns, two regression models were developed. Model one was adjusted for age and sex. Model two was additionally adjusted for marital status, SEIFA, smoking status, alcohol risk and PAL. The trend of association was assessed using the quintiles of dietary patterns as continuous variables. A restricted cubic spline regression analysis was performed to determine the dose–response relationship between dietary constructs and the risk of obesity. All analyses were conducted using STATA/SE version 16 (Stata, StataCorp LP, College Station, TX, USA).

## 3. Results

In this study, 787 non-obese participants of the NWAHS cohort from a total of 2500 subjects at Stage 3 were included (Figure 1). A total of 1326 (53%) participants were excluded because: (1) there was invalid baseline energy intake (*n* = 177); (2) there were missing values for BMI at Stage 3 or NW15 (*n* = 1074); (3) the participants were obese at Stage 3 (*n* = 387); or (4) there were missing values of covariates (*n* = 75). The mean age of participants was 58.7 years (SD 12.9) and 45.9% were men. The mean BMI at baseline was 25.6 kg/m^2^ (SD 2.7) and the incidence of obesity was 7.62% at the 5-year follow up.

Characteristics of the participants at baseline according to the extreme quintiles of DII, diet quality, PDI, hPDI and uPDI are shown in Table 1. The remaining data are provided in Supplementary Tables S2 and S3, respectively. The overall mean DII score of the population was 1.43 (1.36). Diet quality, prudent pattern, PDI and hPDI were inversely associated with the DII, whereas the Western pattern and uPDI were positively associated with the DII. Based on BMI, trends for all diet factors were significant. The prudent pattern, PDI and hPDI were positively associated with diet quality. Conversely, an inverse association was observed between diet quality with the Western pattern and uPDI. No significant trend was observed between quintiles of diet indices and SEIFA. However, around half of the participants were in the low-income group and did not engage in sufficient physical activity.

### 3.1. Anti-Inflammatory Diet, Prudent Pattern, and hPDI Were Inversely Associated with a Lower Risk of Obesity

In model 2, a significant inverse trend was found between prudent dietary pattern (RRQ5VsQ1 = 0.38; 95% CI: 0.15–0.96); *p* = 0.013), diet quality (RRQ5VsQ1 = 0.23; 95% CI: 0.08–0.66); *p* =0.006), and hPDI (RRQ5VsQ1 = 0.31; 95% CI: 0.12–0.77); *p* = 0.006) with the risk of obesity (Table 2). PDI (RRQ5 vs. Q1 = 0.56; 95% CI: 0.23–1.33); *p* =0.19) also showed an association with a reduced risk of obesity. For the DII, a more anti-inflammatory diet (RRQ2 vs. Q1 = 0.58; 95% CI: 0.20–1.68); *p* = 0.06) was associated with a lower risk of obesity. Conversely, the higher quintiles, representing a more pro-inflammatory diet, were associated with a higher risk of obesity. In addition, diet quality displayed a stronger association with a lower risk of obesity compared to other diet factors.

### 3.2. Western Dietary Pattern and uPDI Were Associated with a Higher Risk of Obesity

A Western pattern (RRQ5 vs. Q1 = 1.13; 95% CI: 0.51–2.53); *p* = 0.872) and uPDI (RRQ5 vs. Q1 = 1.74; 95% CI: 0.74–4.11); *p* = 0.134) were associated with the risk of obesity (Model 1) although no significant trend was obtained. After adjustment for other covariates (Model 2), the trend was unchanged; Western pattern (RRQ5 vs. Q1 = 2.16; 95% CI: 0.76–6.08); *p* = 0.167) and uPDI (RRQ5 vs. Q1 = 1.94; 95% CI: 0.81–4.66); *p* = 0.088) (Table 2). However, the association between Western dietary pattern and the risk of obesity was stronger after adjustment for covariates.

### 3.3. Diet and Risk of Obesity Dose–Response Relationship

**Figure 2:**
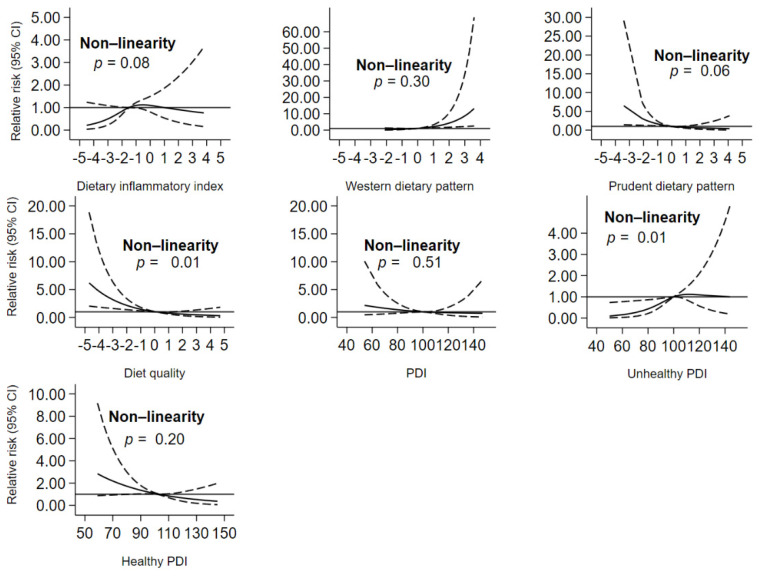
Dose–response relationship curve showing the relationship between dietary constructs and the risk of obesity. PDI: Plant-Based Dietary Index.

We found a significant non-linear association between diet quality and an unhealthy plant-based diet and the risk of obesity (*p* value non-linear <0.05) (Figure 2).

## 4. Discussion

To the best of our knowledge, this study is the first to investigate the association between a number of dietary indices and patterns (both a priori and a posteriori dietary data analysis methods) with incidence of obesity in a community-based cohort. Our study found that the incidence of obesity, in the NWAHS cohort, between 2010 and 2015 was 7.62%. After adjustment for potential confounders, an anti-inflammatory diet (a lower DII score), diet quality, prudent dietary pattern, overall PDI and hPDI were associated with a lower risk of obesity. Conversely, a pro-inflammatory diet (a higher DII score), Western dietary pattern, and uPDI were related to a higher risk of obesity. The association between diet quality and unhealthy PDI with the risk of obesity were non-linear.

### 4.1. DII and the Development of Obesity

Several studies have assessed the relationship between the inflammatory potential of diet and overweight or obesity. A higher DII score, indicating a more pro-inflammatory diet, has been associated with a higher BMI, waist circumference, waist-height ratio, and increased risk of cardiovascular diseases and cancer [15,28,29,30]. A cross-sectional study, in the Cohort of Universities of Minas Gerais, also displayed an increased prevalence of obesity with a higher DII score [31]. Furthermore, a longitudinal study in a Mediterranean cohort also showed that a greater DII score was associated with an increased average yearly weight change and the incidence of overweight and obesity over 10 years follow up [32]. Our findings are in alignment with these previous studies in confirming that a higher DII score was associated with an increased risk of obesity while a lower DII score was related to a lower risk of obesity.

### 4.2. Dietary Patterns and the Risk of Obesity

The current food-based dietary recommendations highlight a healthy diet, characterized by increased intake of fruits and vegetables and reduced consumption of high fat foods, for better health. In agreement with the recommendations, our findings indicate that adherence to a prudent diet was associated with a lower risk of obesity. Outcomes from meta-analyses showed that the prudent dietary pattern was associated with a lower risk of overweight or obesity [33,34]. Another study also has shown that a higher adherence to a prudent dietary pattern was related with a lower risk of central obesity, abnormal glucose level and metabolic syndrome [9]. Furthermore, intake of reduced-fat dairy products and high-fibre foods, which is aligned with food groups identified in the prudent dietary pattern in the current cohort [23], was related with a reduced increase in waist circumference in both women and men [35].

Our findings also revealed an association between the Western dietary pattern and the risk of obesity. Although there are inconsistent findings on the relationship between food patterns and BMI or obesity [36,37], the majority of studies have revealed a positive relationship between a Western dietary pattern and the risk of obesity, central obesity, and higher body fat proportion [38,39].

### 4.3. Plant-Based Diet and the Risk of Obesity

Healthy dietary patterns (e.g., Mediterranean, Dietary Approaches to Stop Hypertension (DASH), Nordic, vegan, vegetarian), which emphasize an intake of plant-based foods, have been associated with weight loss [13] and a lower-risk of obesity-related chronic diseases [11]. Our study showed that overall PDI was related to a lower risk ratio of obesity, suggesting that consumption of more plant-based foods and limiting animal-based diets may reduce the risk of obesity. The relationship between adherence to a plant-based diet, or limiting the intake of animal-based foods, with the risk of obesity is still under debate [40,41]. Nevertheless, evidence has shown that increased consumption of animal foods was associated with a greater odds of obesity [38] and adherence to a vegan or vegetarian diet was related to a lower BMI [40]. However, these results were mainly obtained from cross-sectional studies which are prone to information bias [40]. Thus, more prospective studies are required to confirm this relationship.

In addition to the overall PDI, we also examined the association between hPDI or uPDI with the risk of obesity. Our finding showed that uPDI was associated with a greater risk of obesity while an inverse association was observed for hPDI. This suggests a protective effect of healthy plant-based foods against weight gain. Healthy plant-based foods (e.g., whole grains, fruits, vegetables, nuts, legumes, and vegetable oils) were related to lower adiposity and fatty liver content [42] and risk of cardiovascular diseases [8]. Importantly, our results are in agreement with three other prospective cohort studies showing that diets rich in healthy plant-based foods were associated with lower weight gain over four years follow up [24].

### 4.4. Potential Mechanisms

Inflammation is the substrate on which several putative mechanisms can work to increase obesity. Prominent among these is insulin resistance [43]. Although inflammation is often considered a consequence of the obese state, increasing evidence suggest that inflammation may contribute to the development of obesity. For instance, knockout animal studies have implicated pro-inflammatory cytokines, such as IL-6 and TNF-α, in the development of obesity [44,45]. Furthermore, cohort studies in middle-aged and older adults have shown that elevated levels on inflammatory markers, such as inflammation-sensitive proteins (i.e., fibrinogen, haptoglobin, alpha1-antitrypsin, orosomucoid, and ceruloplasmin), IL-6, albumin, C-reactive protein (CRP), factor VIIIc, white blood cell count, and platelet count, were associated with a greater risk of future weight gain [46,47,48].

There are several ways that diet can influence inflammation. First, diet modifies oxidative stress levels in the body, leading to changes in inflammatory status. For instance, intake of a high-fat diet triggers activation of pro-inflammatory pathways, causing oxidative stress that leads into systemic inflammation [49]. On the other hand, intake of fruits and vegetables lowers oxidative stress and subsequently, inflammation [50,51]. Fruits and vegetables are rich in antioxidants, which play a key role in reducing oxidative stress through its antioxidative capacity. Studies have linked high antioxidative scores, measured using oxygen radical absorbance capacity (ORAC), to anti-inflammatory diet [52] and to a reduced level of clinical inflammatory markers, such as IL-6 [53] and CRP [53,54]. Therefore, this may explain how adherence to an anti-inflammatory, healthy and plant-based diet, which are all characterized by an increased intake of fruits and vegetables, reduce the risk of obesity.

Second, diet can alter gut microbiome composition which, in turn, can affect inflammation. The type of diet influences the characteristics of gut microbiota/microbiome [55,56,57], in particular the balance of pro- and anti-inflammatory gut microbiota population in the gut [58]. The Western diet has been associated with an increased pro-inflammatory potential of gut microbiota [59]. Conversely, the plant-based diet has been linked with producers of short chain fatty acids (SCFA) [60], by-products of gut microbiota that have anti-inflammatory properties [61]. Furthermore, a study has also shown that a lower DII score was associated with anti-inflammatory gut microbiota and reduced inflammatory markers [52,62]. Altogether, this may explain the protective effect of an anti-inflammatory, healthy and plant-based diet towards obesity by preventing inflammation through gut microbiota. Further studies, however, are still required to confirm the current potential mechanisms and to reveal other possible mechanisms underlying the association between diet, inflammation and obesity.

### 4.5. Strengths and Limitations

There are a number of limitations of this study, including the possibility of errors in self-report of diet using the FFQ and exclusion of certain food groups that are simply left off the food list [63]. However, the FFQ has been widely used to construct DII [32], dietary patterns [23], and PDI [8] for cohort studies. Thus, this validating evidence provides some confidence about the reliability of the FFQ to assess overall dietary intake. There are also nutrients and/or foods, such as sodium [64], calcium [65], potassium [66], riboflavin (vitamin B2) [67], and pantothenic acid (vitamin B5) [68], which were not incorporated in the DII that may have impacted on inflammation or the risk of obesity. However, none of these parameters had sufficient evidence to warrant inclusion in the DII formulation. In addition, the BMI at NW15 was calculated based on self-reported height and weight data. Although the self-reported method has been previously validated, this may have underestimated the risk of obesity as an outcome [69]. Furthermore, the sample size in this study was relatively small, which may have contributed to less precise estimates (wider confidence intervals, which included the null value) despite large effect sizes (point estimates). A strength of this study is that it provides comprehensive analysis using multiple dietary indices and a posteriori dietary patterns to determine the longitudinal relationship between diet and the risk of obesity.

### 4.6. Significance

Results from this study support the notion that diet is pivotal in obesity prevention, where the intake of an anti-inflammatory diet and healthy plant-based food, as well as following a healthy dietary pattern, may protect individuals against obesity. This may contribute to improved dietary recommendations and could increase public awareness to adhere to a healthy diet, particularly among low-income individuals and those with insufficient physical activity. Future cohort studies involving a larger number of participants and a longer follow up would be required to confirm this longitudinal association.

## 5. Conclusions

Intake of an anti-inflammatory diet, healthy diet and healthy plant-based foods was associated with a lower risk of obesity. Public health messages should target low-income individuals and those with insufficient physical activity with messaging to increase adherence to plant-based, anti-inflammatory diets and increase physical activity [70,71].

## Figures and Tables

**Table 1:** Baseline characteristics of participants based on the highest and lowest quintiles of DII, diet quality, PDI, uPDI and hPDI.

Characteristics	Overall	DII		*p*- Trend	Dietary Quality		*p*- Trend	PDI		*p*- Trend	uPDI		*p*- Trend	hPDI		*p*-Trend
		Q1	Q5		Q1	Q5		Q1	Q5		Q1	Q5		Q1	Q5	
Sex (*n*,%)																
Male	361 (45.9)	59 (16.3)	91 (25.2)	0.001	116 (32.1)	34 (9.4)	<0.001	82 (22.7)	74 (20.5)	0.26	67 (18.6)	73 (20.2)	0.20	100 (27.7)	51 (14.1)	<0.001
Female	426 (54.1)	99 (23.2)	66 (15.5)		42 (9.9)	123 (28.9)		82 (19.3)	79 (18.5)		103 (24.2)	77 (18.1)		68 (16.0)	104 (24.4)	
Age (mean, SD), year	58.7 (12.9)	59.7 (10.8)	56.7 (13.2)	0.004	57.0 (12.7)	60.3 (11.3)	0.004	58.6 (13.7)	59.9 (13.3)	0.001	61.4 (11.0)	56.6 (13.6)	0.01	54.9 (13.5)	62.7 (11.4)	0.03
BMI (mean, SD), kg/m^2^	25.6 (2.7)	25.3 (2.7)	25.8 (2.8)	0.004	25.9 (2.7)	24.9 (2.9)	<0.001	26.3 (2.4)	25.4 (2.8)	0.01	25.4 (2.8)	25.6 (3.0)	0.002	25.8 (2.9)	25.0 (2.7)	0.02
Educational Status (*n*,%)																
Did not complete high school / high school level	383 (48.7)	70 (18.3)	81 (21.2)	0.02	87 (22.7)	71 (18.5)	0.004	93 (24.3)	64 (16.7)	0.01	80 (20.9)	84 (21.9)	0.33	80 (20.9)	67 (17.5)	0.64
Trade / certificate / diploma	252 (32.0)	52 (20.6)	61 (24.2)		55 (21.8)	42 (16.7)		53 (21.0)	47 (18.7)		57 (22.6)	48 (19.1)		58 (23.0)	50 (19.8)	
Degree or higher	152 (19.3)	36 (23.7)	15 (9.9)		16 (10.5)	44 (29.0)		18 (11.8)	42 (27.6)		33 (21.7)	18 (11.8)		30 (19.7)	38 (25.0)	
Marital Status (*n*,%)																
Married/living with partner	572 (72.7)	121 (21.2)	103 (18.0)	0.09	114 (19.9)	109 (19.1)	0.13	116 (20.3)	113 (19.8)	0.53	117 (20.5)	117 (20.5)	0.47	130 (22.7)	99 (17.3)	<0.001
Separated / divorced	101 (12.8)	21 (20.8)	30 (29.7)		23 (22.7)	23 (22.7)		30 (29.7)	13 (12.9)		30 (29.7)	15 (14.9)		20 (19.8)	20 (19.8)	
Widowed	72 (9.2)	13 (18.1)	14 (19.4)		12 (16.7)	20 (27.8)		12 (16.7)	17 (23.6)		16 (22.2)	11 (15.3)		7 (9.72)	30 (41.7)	
Never married	41 (5.2)	3 (7.3)	10 (24.4)		9 (22.0)	5 (12.2)		5 (12.2)	10 (24.4)		6 (14.6)	7 (17.1)		11 (26.8)	6 (14.6)	
Not stated	1 (0.1)	0 (0.0)	0 (0.0)		0 (0.0)	0 (0.0)		1 (100.0)	0 (0.0)		1 (100.0)	0 (0.0)		0 (0.0)	0 (0.0)	
Smoking Status (*n*,%)																
Non-smoker	388 (49.3)	87 (22.4)	57 (14.7)	0.002	57 (14.7)	86 (22.2)	<0.001	70 (18.0)	95 (24.5)	0.003	90 (23.2)	75 (19.3)	0.78	78 (20.1)	86 (22.2)	0.15
Ex-smoker	320 (40.7)	62 (19.4)	71 (22.2)		71 (22.2)	66 (20.6)		73 (22.8)	52 (16.3)		67 (20.9)	59 (18.4)		67 (20.9)	62 (19.4)	
Current smoker	79 (10.0)	9 (11.4)	29 (36.7)		30 (38.0)	5 (6.3)		21 (26.6)	6 (7.6)		13 (16.5)	16 (20.3)		23 (29.1)	7 (8.9)	
SEIFA (*n*, %)																
Lowest quintile	174 (22.1)	29 (16.7)	41 (23.6)	0.09	46 (26.4)	30 (17.2)	0.28	38 (21.8)	33 (19.0)	0.86	28 (16.1)	38 (21.8)	0.49	42 (24.1)	28 (16.1)	0.84
Low quintile	187 (23.8)	45 (24.1)	44 (23.5)		44 (23.5)	35 (18.7)		42 (22.5)	28 (15.0)		46 (24.6)	42 (22.5)		40 (21.4)	33 (17.7)	
Middle quintile	165 (21.0)	31 (18.8)	28 (17.0)		28 (17.0)	34 (20.6)		31 (18.8)	33 (20.0)		38 (23.0)	23 (13.9)		35 (21.2)	31 (18.8)	
High quintile	204 (25.9)	39 (19.1)	37 (18.1)		34 (16.7)	43 (21.1)		42 (20.6)	50 (24.5)		46 (22.6)	40 (19.6)		42 (20.6)	50 (24.5)	
Highest quintile	57 (7.2)	14 (24.6)	7 (12.3)		6 (10.5)	15 (26.3)		11 (19.3)	9 (15.8)		12 (21.1)	7 (12.3)		9 (15.8)	13 (22.8)	
Alcohol Risk (*n*,%)																
Non-drinkers and no risk	377 (47.9)	72 (19.1)	72 (19.1)	0.30	94 (24.9)	57 (15.1)	<0.001	68 (18.0)	82 (21.8)	0.16	76 (20.2)	82 (21.8)	0.48	86 (22.8)	73 (19.4)	0.91
Low risk	318 (40.4)	71 (22.3)	58 (18.2)		35 (11.0)	84 (26.4)		66 (20.8)	59 (18.6)		74 (23.3)	49 (15.4)		60 (18.9)	65 (20.4)	
Intermediate risk	20 (2.5)	3 (15.0)	5 (25.0)		10 (50.0)	2 (10.0)		9 (45.0)	1 (5.0)		4 (20.0)	2 (10.0)		4 (20.0)	2 (10.0)	
High to very high risk	8 (1.0)	0 (0.0)	2 (25.0)		3 (37.5)	0 (0.0)		3 (37.5)	0 (0.0)		2 (25.0)	3 (37.5)		2 (25.0)	1 (12.5)	
Incomplete information	64 (8.1)	12 (18.8)	20 (31.3)		16 (25.0)	14 (21.9)		18 (28.1)	11 (17.2)		14 (21.9)	14 (21.9)		16 (25.0)	14 (21.9)	
PAL (*n*,%)																
No activity	101 (12.8)	8 (7.9)	25 (24.6)	<0.001	25 (24.8)	7 (6.9)	<0.001	27 (26.7)	13 (12.9)	0.32	17 (16.8)	29 (28.7)	0.003	21 (20.8)	16 (15.8)	0.01
Activity but not sufficient	322 (40.9)	60 (18.6)	78 (24.2)		75 (23.3)	58 (18.0)		61 (18.9)	64 (19.9)		59 (18.3)	73 (22.7)		82 (25.5)	49 (15.2)	
Sufficient activity	364 (46.3)	90 (24.7)	54 (14.5)		58 (15.9)	92 (25.3)		76 (20.9)	76 (20.9)		94 (25.8)	48 (13.2)		65 (17.9)	90 (24.7)	
DII (mean, SD)	−1.43 (1.36)				−0.03 (1.25)	−2.64 (0.82)	0.43	−0.64 (1.42)	−2.24 (0.99)	0.18	−2.11 (1.06)	−0.57 (1.50)	0.15	−0.30 (1.34)	−2.27 (1.05)	0.21
Prudent DP (mean, SD)	0.13 (1.02)	1.11 (0.97)	−0.77 (0.70)	0.39	−0.78 (0.70)	1.37 (0.83)	0.40	−0.66 (0.73)	0.92 (0.92)	0.30	1.05 (0.99)	−0.71 (0.77)	0.33	−0.44 (0.88)	0.77 (1.11)	0.14
Western DP (mean, SD)	−0.06 (0.93)	−0.46 (0.79)	0.38 (1.05)	0.10	1.02 (0.90)	−0.75 (0.63)	0.30	−0.19 (1.05)	0.17 (0.91)	0.01	−0.03 (0.93)	−0.14 (0.86)	<0.001	0.69 (0.97)	−0.67 (0.70)	0.25
Dietary quality (mean, SD)	0.18 (1.43)	1.56 (1.12)	−1.15 (1.30)	0.42				−0.47 (1.43)	0.75 (1.36)	0.10	1.09 (1.37)	−0.56 (1.23)	0.15	−1.12 (1.30)	1.44 (1.18)	0.35
PDI (mean, SD)	101.8 (12.8)	108.9 (12.5)	91.7 (10.6)	0.19	95.6 (12.1)	108.3 (12.1)	0.095				104.1 (11.6)	98.5 (12.5)	0.02	94.0 (10.9)	109.5 (12.5)	0.14
uPDI (mean, SD)	99.8 (14.3)	92.3 (12.9)	108.4 (13.4)	0.14	107.1 (14.4)	90.4 (11.5)	0.17	101.9 (13.7)	97.7 (12.6)	0.02				107.8 (14.3)	91.8 (12.1)	0.14
hPDI (Mean, SD)	103.1 (14.7)	113.9 (12.5)	91.7 (14.0)	0.26	88.3 (12.1)	116.0 (12.6)	0.39	95.9 (14.9)	112.1 (12.5)	0.15	110.4 (14.4)	94.5 (14.1)	0.14			

BMI: body mass index; SEIFA: Socio-Economic Indexes for Areas; PAL: physical activity level; DII: dietary inflammatory index; DP: dietary pattern; PDI: plant-based diet; uPDI: unhealthy plant-based diet; hPDI: healthy plant-based diet. *p*-trend was calculated considering all quartiles (Q1 to Q5) of the dietary constructs.

**Table 2:** Multivariable adjusted models (95% confidence intervals) for the risk of obesity.

Model	Relative Risk (95% CI)					*p*-Trend
	Q1	Q2	Q3	Q4	Q5	
Dietary inflammatory index						
Model 1	1.00	0.56 (0.19–1.62)	1.52 (0.68–3.41)	1.62 (0.73–3.61)	1.78 (0.81–3.91)	**0.03**
Model 2	1.00	0.58 (0.20–1.68)	1.64 (0.73–3.68)	1.72 (0.78–3.81)	1.59 (0.72–3.50)	0.06
Prudent pattern						
Model 1	1.00	0.70 (0.37–1.33)	0.55 (0.30–1.11)	0.34 (0.15–0.79)	0.36 (0.16–0.83)	**0.002**
Model 2 *	1.00	0.75 (0.39–1.43)	0.58 (0.28–1.21)	0.39 (0.16–0.94)	0.38 (0.15–0.96)	**0.01**
Western pattern						
Model 1	1.00	0.96 (0.43–2.16)	1.14 (0.52–2.47)	0.82 (0.35–1.92)	1.13 (0.51–2.53)	0.87
Model 2 *	1.00	1.36 (0.59–3.12)	1.77 (0.77–4.07)	1.57 (0.59–4.16)	2.16 (0.76–6.08)	0.17
Diet quality						
Model 1	1.00	0.72 (0.37–1.37)	0.46 (0.21–0.99)	0.62 (0.30–1.26)	0.26 (0.10–0.70)	**0.007**
Model 2 *	1.00	0.70 (0.36–1.35)	0.40 (0.17–0.90)	0.54 (0.25–1.19)	0.23 (0.08–0.66)	**0.006**
Plant-based dietary index						
Model 1	1.00	0.76 (0.38–1.51)	0.84 (0.42–1.67)	0.65 (0.31–1.38)	0.45 (0.19–1.05)	0.07
Model 2	1.00	0.75 (0.37–1.50)	0.87 (0.44–1.72)	0.68 (0.32–1.45)	0.56 (0.23–1.33)	0.19
Healthy plant-based dietary index						
Model 1	1.00	0.35 (0.16–0.77)	0.73 (0.38–1.39)	0.48 (0.23–0.99)	0.30 (0.12–0.74)	**0.02**
Model 2	1.00	0.367 (0.17–0.80)	0.67 (0.35–1.29)	0.39 (0.19–0.81)	0.31 (0.12–0.77)	**0.006**
Unhealthy plant-based dietary index						
Model 1	1.00	1.30 (0.52–3.21)	1.92 (0.84–4.42)	1.88 (0.81–4.37)	1.74 (0.74–4.11)	0.13
Model 2	1.00	1.33 (0.54–3.28)	1.95 (0.85–4.49)	1.87 (0.81–4.33)	1.94 (0.81–4.66)	0.09

Model 1 was adjusted for sex and age. Model 2 was additionally adjusted for educational status, marital status, SEIFA, smoking status, alcohol risk, and PAL. * was additionally adjusted for total energy intake. Total energy intake was adjusted in the dietary constructs for the dietary inflammatory index, plant-based dietary index, healthy and unhealthy plant-based dietary index. Statistically significant values (*p* < 0.05) are indicated in bold.

## Data Availability

The data presented in this study are available on request from the North West Adelaide Health Study Chief Investigators. The data are not publicly available as this is an on going cohort study.

## Supplementary Materials

The following are available online at https://www.mdpi.com/article/10.3390/nu13051536/s1, Table S1: Food items components for the overall PDI, uPDI and hPDI., Table S2: Baseline characteristics of participants based on quintiles of prudent and Western dietary pattern, Table S3: Baseline characteristics of participants based on quintiles 2–4 of DII, diet quality, PDI, uPDI and hPDI.
