# Supplementary material for: Association between Dietary Inflammatory Index, Dietary Patterns, Plant-Based Dietary Index and the Risk of Obesity
Source: Nutrients. 2021 May 2;13(5):1536. doi: 10.3390/nu13051536 (PMC8147427; doi:10.3390/nu13051536)
Supplement: Supplementary file 1 [file nutrients-13-01536-s001.zip › Supplementary Tables_Nutrients.pdf]

Supplementary Table 1. Food items components for the overall PDI, uPDI and hPDI.

**Plant food groups**	
Healthy	
Whole grains	Whole-grain bread, rye bread, crispbread, oatmeal, whole-grain breakfast cereal
Fruits	Apple, pear, orange, mandarin, kiwi, banana, plum, peach, apricot, strawberry, currant, blueberry, blackberry, grapes, mixed fruits
Vegetables	Carrot, tomato, lettuces, cabbage, cucumber, pepper, garlic, sauerkraut, spinach, cauliflower, broccoli, kohlrabi, asparagus, zucchini, mixed vegetables
Nuts	Nuts, seeds
Legumes	Beans, peas
Vegetable oils	Olive oil, sunflower oil, linseed oil, thistle oil
Tea and coffee	Tea, coffee, decaffeinated coffee
Less healthy	
Fruit juices	Orange juice, apple juice, currant juice, grape juice, cherry juice, pineapple juice, multivitamin juice
Refined grains	White bread, toast, rice, pasta, pancakes
Potatoes	Potatoes, potato products
Sugar-sweetened beverages	Lemonade, soft drinks
Sweets and desserts	Sugar, honey, jams and jellies, chocolates, candies, sweets without chocolate, syrup, sorbets, cake, cookies, biscuits
**Animal food groups**	
Animal fat	Butter, fish oil, other animal fat
Dairy	Milk, mixed milk drinks, cream, yogurt, curd, cheese, cream cheese, ice cream
Egg	Eggs
Fish or seafood	Fish, seafood
Meat	Beef, calf, pork, lamb, horse, goat, poultry, rabbit, game, processed meat
Miscellaneous animal-based foods	Casserole, mayonnaise or creamy salad dressing, pizza

Supplementary Table 2. Baseline characteristics of participants based on quintiles of prudent and western dietary pattern.

Characteristics	Prudent DP					p-value for trend	Western DP					p-value for trend
	Q1	Q2	Q3	Q4	Q5		Q1	Q2	Q3	Q4	Q5	
**Sex (n, %)**												
Male	101 (28.0)	76 (21.1)	61 (16.9)	65 (18.0)	58 (16.1)	< 0.001	36 (10.0)	52 (14.4)	63 (17.5)	94 (26.0)	116 (32.1)	< 0.001
Female	57 (13.4)	81 (19.0)	97 (22.8)	92 (21.6)	99 (23.2)		122 (28.6)	105 (24.7)	95 (22.3)	63 (14.8)	41 (9.6)	
**Age (mean, SD), year**	58.6 (13.2)	59.3 (13.5)	57.7 (13.1)	57.2 (12.3)	60.8 (12.0)	< 0.001	59.4 (12.5)	57.9 (12.4)	60.8 (12.8)	60.0 (12.6)	55.4 (13.5)	0.004
**BMI (mean, SD), kg/m^2**	26.2 (2.6)	25.8 (2.6)	25.5 (2.9)	25.4 (2.7)	25.3 (2.7)	0.015	25.5 (2.7)	25.2 (2.7)	25.7 (2.83)	25.9 (2.7)	25.9 (2.7)	0.006
**Educational status (n, %)**												
Did not complete high school / high school level	91 (23.8)	79 (20.6)	82 (21.4)	70 (18.3)	61 (15.9)	< 0.001	78 (20.4)	74 (19.3)	78 (20.4)	73 (19.1)	80 (20.9)	0.198
Trade / certificate / diploma	56 (22.2)	49 (19.4)	49 (19.4)	48 (19.1)	50 (19.8)		50 (19.8)	43 (17.1)	54 (21.4)	48 (19.1)	57 (22.6)	
Degree or higher	11 (7.2)	29 (19.1)	27 (17.8)	39 (25.7)	46 (30.3)		30 (19.7)	40 (26.3)	26 (17.1)	36 (23.7)	20 (13.2)	
**Marital status (n, %)**												
Married or living with partner	99 (17.3)	118 (20.6)	121 (21.2)	123 (21.5)	111 (19.4)	0.082	102 (17.8)	117 (20.5)	119 (20.8)	120 (21.0)	114 (19.9)	0.163
Separated / divorced	29 (28.7)	19 (18.8)	17 (16.8)	11 (10.9)	25 (24.8)		31 (30.7)	16 (15.8)	18 (17.8)	16 (15.8)	20 (19.8)	
Widowed	20 (27.8)	13 (18.1)	9 (12.5)	14 (19.4)	16 (22.2)		19 (26.4)	15 (20.8)	15 (20.8)	14 (19.4)	9 (12.5)	
Never married	10 (24.4)	7 (17.1)	11 (26.8)	8 (19.5)	5 (12.2)		6 (14.6)	9 (22.0)	6 (14.6)	7 (17.1)	13 (31.7)	
Not stated	0 (0.0)	0 (0.0)	0 (0.0)	1 (100.0)	0 (0.0)		0 (0.0)	0 (0.0)	0 (0.0)	0 (0.0)	1 (100.0)	
**SEIFA (n, %)**												
Lowest quintile	45 (25.9)	30 (17.2)	37 (21.3)	35 (20.1)	27 (15.5)	0.081	30 (17.2)	35 (20.1)	35 (20.1)	30 (17.2)	44 (25.3)	0.528
Low quintile	45 (24.1)	42 (22.5)	27 (14.4)	35 (18.7)	38 (20.3)		40 (21.4)	30 (16.0)	44 (23.5)	38 (20.3)	35 (18.7)	
Middle quintile	21 (12.7)	37 (22.4)	43 (26.1)	28 (17.0)	36 (21.8)		31 (18.8)	34 (20.6)	31 (18.8)	39 (23.6)	30 (18.2)	
High quintile	40 (19.6)	38 (18.6)	40 (19.6)	46 (22.6)	40 (19.6)		42 (20.6)	43 (21.1)	43 (21.1)	39 (19.1)	37 (18.1)	
Highest quintile	7 (12.3)	10 (17.5)	11 (19.3)	13 (22.8)	16 (28.1)		15 (26.3)	15 (26.3)	5 (8.8)	11 (19.3)	11 (19.3)	
**Smoking status (n, %)**												
Non smoker	66 (17.0)	66 (17.0)	78 (20.1)	91 (23.5)	87 (22.4)	< 0.001	75 (19.3)	83 (21.4)	85 (21.9)	84 (21.7)	61 (15.7)	0.012
Ex-smoker	64 (20.0)	72 (22.5)	61 (19.1)	59 (18.4)	64 (20.0)		73 (22.8)	57 (17.8)	57 (17.8)	63 (19.7)	70 (21.9)	
Current smoker	28 (35.4)	19 (24.1)	19 (24.1)	7 (8.9)	6 (7.6)		10 (12.7)	17 (21.5)	16 (20.3)	10 (12.7)	26 (32.9)	
**Alcohol risk (n, %)**												
Non-drinkers and no risk	87 (23.1)	75 (19.9)	74 (19.6)	73 (19.4)	68 (18.0)	0.044	55 (14.6)	65 (17.2)	68 (18.0)	91 (24.1)	98 (26.0)	< 0.001
Low risk	46 (14.5)	59 (18.6)	68 (21.4)	70 (22.0)	75 (23.6)		80 (25.2)	78 (24.5)	71 (22.3)	55 (17.3)	34 (10.7)	
Intermediate risk	8 (40.0)	7 (35.0)	2 (10.0)	1 (5.0)	2 (10.0)		4 (20.0)	2 (10.0)	5 (25.0)	1 (5.0)	8 (40.0)	
High to very high risk	3 (37.5)	1 (12.5)	3 (37.5)	0 (0.0)	1 (12.5)		2 (25.0)	0 (0.0)	2 (25.0)	1 (12.5)	3 (37.5)	
Incomplete information	14 (21.9)	15 (23.4)	11 (17.2)	13 (20.3)	11 (17.2)		17 (26.6)	12 (18.8)	12 (18.8)	9 (14.1)	14 (21.9)	
**PAL (n, %)**												
No activity	33 (32.7)	28 (27.7)	20 (19.8)	10 (9.9)	10 (9.9)	< 0.001	9 (8.9)	24 (23.8)	28 (27.7)	16 (15.8)	24 (23.8)	0.015
Activity but not sufficient	72 (22.4)	59 (18.3)	68 (21.1)	64 (19.9)	59 (18.3)		61 (18.9)	59 (18.3)	61 (18.9)	76 (23.6)	65 (20.2)	
Sufficient activity	53 (14.6)	70 (19.2)	70 (19.2)	83 (22.8)	88 (24.2)		88 (24.2)	74 (20.3)	69 (19.0)	65 (17.9)	68 (18.7)	
**DII (mean, SD)**	-0.01 (1.23)	-1.07 (1.13)	-1.56 (1.00)	-1.94 (0.97)	-2.57 (0.89)	0.390	-2.02 (1.17)	-1.59 (1.24)	-1.49 (1.35)	-1.29 (1.18)	-0.74 (1.50)	0.089
**Prudent DP (mean, SD)**							0.22 (1.06)	0.05 (0.90)	0.14 (0.99)	0.20 (0.99)	0.01 (1.11)	0.001
**Western DP (mean, SD)**	0.10 (1.08)	-0.15 (0.84)	-0.04 (0.88)	-0.07 (0.85)	-0.12 (0.96)	0.003						
**Dietary quality (mean, SD)**	-1.27 (1.22)	-0.33 (0.85)	0.07 (0.88)	0.69 (0.87)	1.76 (1.15)	0.493	1.40 (1.13)	0.63 (0.92)	0.31 (1.01)	-0.10 (0.99)	-1.35 (1.38)	0.385
**PDI (mean, SD)**	90.1 (10.6)	98.3 (9.7)	102.1 (9.8)	107.1 (11.3)	111.5 (10.9)	0.327	98.7 (12.9)	100.2 (11.4)	102.9 (13.1)	104.3 (12.3)	103.0 (13.5)	0.020
**uPDI (mean, SD)**	111.7 (13.1)	105.3 (11.2)	99.6 (10.9)	94.9 (11.9)	87.6 (11.3)	0.334	99.5 (14.1)	101.2 (13.3)	99.8 (14.7)	98.4 (14.0)	100.2 (15.4)	<0.001
**hPDI (Mean, SD)**	94.5 (15.0)	101.2 (12.7)	101.8 (12.9)	105.6 (12.8)	112.3 (14.1)	0.148	114.6 (11.9)	106.8 (12.1)	102.6 (12.6)	99.6 (12.1)	91.6 (14.3)	0.263

BMI: body mass index; SEIFA: Socio-Economic Indexes for Areas; PAL: physical activity level; DII: dietary inflammatory index; DP: dietary pattern; PDI: plant-based diet; uPDI: unhealthy plant-based diet; hPDI: healthy plant-based diet.

Supplementary Table 3. Baseline characteristics of participants based on quintiles 2- 4 of DII, diet quality, PDI, uPDI and hPDI.

Characteristics	DII			Diet Quality			PDI			uPDI			hPDI		
	Q2	Q3	Q4	Q2	Q3	Q4	Q2	Q3	Q4	Q2	Q3	Q4	Q2	Q3	Q4
**Sex (n, %)**															
Male	58 (16.1)	75 (20.8)	78 (21.6)	97 (26.9)	61 (16.9)	53 (14.7)	80 (22.2)	61 (16.9)	64 (17.7)	75 (20.8)	81 (22.4)	65 (18.0)	85 (23.6)	59 (16.3)	66 (18.3)
Female	99 (23.2)	83 (19.5)	79 (18.5)	60 (14.1)	97 (22.8)	104(24.4)	85 (20.0)	94 (22.1)	86 (20.2)	82 (19.3)	77 (18.1)	87 (20.4)	84 (19.7)	77 (18.1)	93 (21.8)
**Age (mean, SD), year**	58.9 (12.7)	58.9 (13.3)	59.3 (14.1)	59. 3 (14.1)	58.5 (13.6)	58.4 (12.4)	58.4 (12.6)	58.4 (12.6)	57.9 (12.6)	58.4 (12.1)	59.3 (13.7)	57.5 (13.5)	58.8 (13.0)	58.4 (12.6)	58.9 (12.6)
**BMI (mean, SD), kg/m^2**	25.6 (2.8)	25.8 (2.6)	25.7 (2.9)	26.0 (2.9)	26.0 (2.6)	25.4 (2.4)	25.4 (3.0)	25.8 (2.8)	25.3 (2.5)	25.8 (2.7)	25.5 (2.6)	26.0 (2.7)	26.1 (2.5)	25.9 (2.8)	25.3 (2.6)
**Educational status (n,%)**															
Did not complete high school / high school level	77 (20.1)	75 (19.6)	80 (20.9)	82 (21.4)	76 (19.8)	67 (17.5)	83 (21.7)	71 (18.5)	72 (18.8)	69 (20.1)	77 (19.1)	73 (21.9)	89 (23.2)	65 (17.0)	82 (21.4)
Trade / certificate / diploma	41 (16.3)	55 (21.8)	43 (17.1)	53 (21.0)	50 (19.8)	52 (20.6)	58 (23.0)	51 (20.2)	43 (17.1)	54 (21.4)	45 (17.7)	48 (19.1)	53 (21.0)	45 (17.9)	46 (18.3)
Degree or higher	39 (25.7)	28 (18.4)	34 (22.4)	22 (14.5)	32 (21.1)	38 (25.0)	24 (15.8)	33 (21.7)	35 (23.0)	34 (22.4)	36 (23.7)	31 (20.4)	27 (17.8)	26 (17.1)	31 (20.4)
**Marital status (n, %)**															
Married or living with partner	111 (19.4)	117 (20.5)	121 (21.2)	119 (20.8)	113 (19.8)	111 (19.4)	119 (20.8)	113 (19.8)	111 (19.4)	120 (21.0)	113 (19.8)	105 (18.4)	139 (24.3)	96 (16.8)	108 (18.9)
Separated / divorced	15 (14.9)	25 (24.8)	15 (14.9)	21 (20.8)	19 (18.8)	18 (17.8)	21 (20.8)	19 (18.8)	18 (17.8)	15 (14.9)	21 (20.8)	20 (19.8)	13 (12.9)	22 (21.8)	26 (25.7)
Widowed	15 (20.8)	9 (12.5)	16 (22.2)	16 (22.2)	16 (22.2)	11 (15.3)	16 (22.2)	16 (22.2)	11 (15.3)	15 (20.8)	16 (22.2)	14 (19.4)	10 (13.9)	13 (18.1)	12 (16.7)
Never married	15 (36.6)	7 (17.1)	5 (12.2)	9 (22.0)	7 (17.1)	10 (24.4)	9 (22.0)	7 (17.1)	10 (24.4)	7 (17.1)	8 (19.5)	13 (31.7)	7 (17.1)	4 (9.8)	13 (31.7)
Not stated	1 (100.0)	0 (0.0)	0 (0.0)	0 (0.0)	0(0.0)	0(0.0)	0 (0.0)	0 (0.0)	0 (0.0)	0 (0.0)	0 (0.0)	0 (0.0)	0 (0.0)	1 (100.0)	0 (0.0)
**SEIFA (n, %)**															
Lowest quintile	37 (21.3)	29 (16.7)	38 (21.8)	39 (22.4)	27 (15.5)	32 (18.4)	37 (21.3)	38 (21.8)	28 (16.1)	34 (19.5)	42 (24.1)	32 (18.4)	41 (23.6)	29 (16.7)	34 (19.5)
Low quintile	31 (16.6)	32 (17.1)	35 (18.7)	34 (18.2)	35 (18.7)	39 (20.9)	42 (22.5)	37 (19.8)	38 (20.3)	37 (19.8)	29 (15.5)	33 (17.7)	39 (20.7)	38 (20.3)	37 (19.8)
Middle quintile	41 (24.9)	33 (20.0)	32 (19.4)	35 (21.2)	31 (18.8)	37 (22.4)	35 (21.2)	31 (18.8)	35 (21.2)	34 (20.6)	38 (23.3)	32 (19.4)	34 (20.6)	28 (17.0)	37 (22.4)
High quintile	43 (21.1)	44 (21.6)	41 (20.1)	39 (19.1)	51 (25.0)	37 (18.1)	41 (20.1)	35 (17.2)	36 (17.7)	41 (20.1)	35 (17.2)	42 (20.6)	39 (19.1)	34 (16.7)	39 (19.1)
Highest quintile	5 (8.8)	20 (35.1)	11 (19.3)	10 (17.5)	14 (24.6)	12 (21.1)	10 (17.5)	14 (24.6)	13 (22.8	11 (19.3)	14 (24.6)	13 (22.8)	16 (28.1)	7 (12.3)	12 (21.1)
**Smoking status (n, %)**															
Non smoker	77 (19.9)	88 (22.7)	79 (20.4)	75 (19.3)	83 (21.4)	87 (22.4)	76 (19.6)	75 (19.3)	72 (18.6)	76 (19.6)	79 (20.4)	68 (17.5)	86 (22.2)	70 (18.0)	68 (17.5)
Ex-smoker	66 (20.6)	57 (17.8)	64 (20.0)	62 (19.4)	61 (19.1)	60 (18.8)	67 (20.9)	59 (18.4)	69 (21.6)	68 (21.3)	62 (19.4)	64 (20.0)	65 (20.3)	54 (16.9)	72 (22.5)
Current smoker	14 (17.7)	13 (16.5)	14 (17.7)	20 (25.3)	14 (17.7)	10 (12.7)	22 (27.9)	21 (26.6)	9 (11.4)	13 (16.5)	17 (21.5)	20 (25.3)	18 (22.8)	12 (15.2)	19 (24.1)
**Alcohol risk (n, %)**															
Non-drinkers and no risk	70 (18.6)	82 (21.8)	81 (21.5)	90 (23.9)	66 (17.5)	70 (18.6)	81 (21.5)	82 (21.8)	64 (17.0)	75 (19.9)	80 (21.2)	64 (17.0)	84 (22.3)	65 (17.2)	69 (18.3)
Low risk	72 (22.6)	57 (17.9)	60 (18.9)	49 (15.4)	75 (23.6)	75 (23.6)	68 (21.4)	54 (17.0)	71 (22.3)	62 (19.5)	62 (19.5)	71 (22.3)	63 (19.8)	57 (17.9)	73 (23.0)
Intermediate risk	1 (5.0)	6 (30.0)	5 (25.0)	1 (5.0)	6 (30.0)	1 (5.0)	4 (20.0)	4 (20.0)	2 (10.0)	7 (35.0)	5 (25.0)	2 (10.0)	4 (20.0)	4 (20.0)	6 (30.0)
High to very high risk	1 (12.5)	2 (25.0)	3 (37.5)	2 (25.0)	2 (25.0)	1 (12.5)	1 (12.5)	2 (25.0)	2 (25.0)	1 (12.5)	0 (0.0)	2 (25.0)	3 (37.5)	1 (12.5)	1 (12.5)
Incomplete information	13 (20.3)	11 (17.2)	8 (12.5)	15 (23.4)	9 (14.1)	10 (15.6)	11 (17.2)	13 (20.3)	11 (17.2)	12 (18.8)	11 (17.2)	13 (20.3)	15 (23.4)	9 (14.1)	10 (15.6)
**PAL (n, %)**															
No activity	18 (17.8)	31 (30.7)	19 (18.8)	33 (32.7)	24 (23.8)	12 (11.9)	22 (21.8)	22 (21.8)	17 (16.8)	15 (14.9)	17 (16.8)	23 (22.8)	29 (28.7)	19 (18.8)	16 (15.8)
Activity but not sufficient	67 (20.8)	54 (16.8)	63 (19.6)	64 (19.9)	60 (18.6)	65 (20.2)	78 (24.2)	59 (18.3)	60 (18.6)	64 (19.9)	69 (21.4)	57 (17.7)	70 (21.7)	59 (18.3)	62 (19.3)
Sufficient activity	72 (19.8)	73 (20.2)	75 (20.6)	60 (16.5)	74 (20.3)	80 (22.0)	65 (17.9)	74 (20.3)	73 (20.1)	78 (21.4)	72 (19.8)	72 (19.8)	70 (19.2)	58 (15.9)	81 (22.3)
**DII (mean, SD)**				-0.99 (1.08)	-1.45 (1.02)	-2.04 (0.91)	-1.03 (1.47)	-1.47 (1.17)	-1.85 (1.01)	-1.83 (1.07)	-1.38 (1.35)	-1.15 (1.24)	-1.31 (1.26)	-1.66 (1.15)	-1.72 (1.08)

**Prudent DP (mean, SD)**	0.47 (0.78)	0.08 (0.76)	-0.26 (0.72)	-0.32 (0.66)	-0.03 (0.68)	0.39 (0.66)	-0.23 (0.79)	0.16 (0.95)	0.54 (0.87)	0.33 (0.79)	0.07 (0.86)	-0.24 (0.67)	0.01 (0.88)	0.10 (0.82)	0.24 (0.98)
**Western DP (mean, SD)**	-0.26 (0.84)	-0.08 (0.82)	0.15 (0.88)	0.12 (0.64)	-0.20 (0.66)	-0.47 (0.63)	-0.06 (0.88)	-0.11 (0.93)	-0.07 (0.82)	-0.08 (0.98)	0.03 (0.99)	-0.06 (0.86)	0.13 (0.78)	-0.13 (0.82)	-0.37 (0.70)
**Dietary quality (mean, SD)**	0.73 (1.01)	0.16 (0.93)	-0.41 (1.01)				-0.17 (1.29)	0.27 (1.41)	0.60 (1.25)	0.40 (1.36)	0.04 (1.43)	-0.18 (1.12)	-0.12 (1.08)	0.23 (1.12)	0.61 (0.99)
**PDI (mean, SD)**	91.7 (10.6)	102.4 (11.5)	100.8 (11.3)	100.9 (12.1)	101.0 (12.1)	103.3 (12.3)				103.1 (13.2)	101.5 (13.3)	101.7 (12.8)	101.2 (10.9)	101.2 (13.1)	103.8 (11.8)
**uPDI (mean, SD)**	96.5 (12.0)	99.6 (14.9)	102.4 (13.1)	104.1 (13.8)	101.3 (12.8)	96.2 (12.6)	102.2 (15.2)	99.0 (14.8)	98.2 (14.7)				102.2 (13.7)	99.8 (13.2)	96.9 (12.8)
**hPDI (Mean, SD)**	107.4 (12.2)	103.1 (11.7)	99.1 (13.1)	99.3 (10.5)	103.5 (11.2)	108.3 (10.7)	97.9 (13.0)	104.1 (13.6)	106.3 (13.5)	107.6 (12.5)	101.4 (13.8)	100.3 (13.4)			

BMI: body mass index; SEIFA: Socio-Economic Indexes for Areas; PAL: physical activity level; DII: dietary inflammatory index; DP: dietary pattern; PDI: plant-based diet; uPDI: unhealthy plant-based diet; hPDI: healthy plant-based diet.
